# Elevated methylation levels, reduced expression levels, and frequent contractions in a clinical cohort of *C9orf72* expansion carriers

**DOI:** 10.1186/s13024-020-0359-8

**Published:** 2020-01-30

**Authors:** Jazmyne L. Jackson, NiCole A. Finch, Matthew C. Baker, Jennifer M. Kachergus, Mariely DeJesus-Hernandez, Kimberly Pereira, Elizabeth Christopher, Mercedes Prudencio, Michael G. Heckman, E. Aubrey Thompson, Dennis W. Dickson, Jaimin Shah, Björn Oskarsson, Leonard Petrucelli, Rosa Rademakers, Marka van Blitterswijk

**Affiliations:** 1 Department of Neuroscience, Mayo Clinic, 4500 San Pablo Road, Jacksonville, FL 32224 USA; 2 Department of Cancer Biology, Mayo Clinic, 4500 San Pablo Road, Jacksonville, FL 32224 USA; 3 Division of Biomedical Statistics and Informatics, Mayo Clinic, 4500 San Pablo Road, Jacksonville, FL 32224 USA; 4 Department of Neurology, Mayo Clinic, 4500 San Pablo Road, Jacksonville, FL 32224 USA

**Keywords:** C9orf72, Amyotrophic lateral sclerosis, Motor neuron disease, Repeat expansion disorder, Anticipation, Hypermethylation, Expansion size, Paternal contraction

## Abstract

**Background:**

A repeat expansion in the C9orf72-SMCR8 complex subunit (*C9orf72*) is the most common genetic cause of two debilitating neurodegenerative diseases: amyotrophic lateral sclerosis (ALS) and frontotemporal dementia (FTD). Currently, much remains unknown about which variables may modify these diseases. We sought to investigate associations between *C9orf72* promoter methylation, RNA expression levels, and repeat length, their potential effects on disease features, as well as changes over time and within families.

**Methods:**

All samples were obtained through the ALS Center at Mayo Clinic Florida. Our primary cohort included 75 unrelated patients with an expanded *C9orf72* repeat, 33 patients who did not possess this expansion, and 20 control subjects without neurodegenerative diseases. Additionally, 67 members from 17 independent *C9orf72* families were selected of whom 33 harbored this expansion. Longitudinally collected samples were available for 35 *C9orf72* expansion carriers. To increase our understanding of *C9orf72*-related diseases, we performed quantitative methylation-sensitive restriction enzyme-based assays, digital molecular barcoding, quantitative real-time PCR, and Southern blotting.

**Results:**

In our primary cohort, higher methylation levels were observed in patients with a *C9orf72* repeat expansion than in patients without this expansion (***p*** = 1.7e-13) or in control subjects (***p*** = 3.3e-07). Moreover, we discovered that an increase in methylation levels was associated with a decrease in total *C9orf72* transcript levels (***p*** = 5.5e-05). These findings aligned with our observation that *C9orf72* expansion carriers had lower expression levels of total *C9orf72* transcripts than patients lacking this expansion (***p*** = 3.7e-07) or control subjects (***p*** = 9.1e-05). We also detected an elevation of transcripts containing intron 1a (upstream of the repeat) in patients carrying a *C9orf72* repeat expansion compared to (disease) controls (***p*** ≤ 0.01), an indication of abortive transcripts and/or a switch in transcription start site usage. While methylation and expression levels were relatively stable over time, fluctuations were seen in repeat length. Interestingly, contractions occurred frequently in parent-offspring transmissions (> 50%), especially in paternal transmissions. Furthermore, smaller repeat lengths were detected in currently unaffected individuals than in affected individuals (***p*** = 8.9e-04) and they were associated with an earlier age at collection (***p*** = 0.008).

**Conclusions:**

In blood from *C9orf72* expansion carriers, we found elevated methylation levels, reduced expression levels, and unstable expansions that tend to contract in successive generations, arguing against anticipation.

## Background

Since the discovery of a repeat expansion in the C9orf72-SMCR8 complex subunit (*C9orf72*) [1, 2], researchers have worked diligently to unravel the mechanisms underlying *C9orf72*-related diseases, including amyotrophic lateral sclerosis (ALS) and frontotemporal dementia (FTD). ALS is a neurodegenerative disorder of the upper and lower motor neurons that results in progressive muscle weakness, often leading to respiratory failure within three to five years after the onset of symptoms. FTD affects the frontal and temporal lobes, causing dementia characterized by changes in personality, behavior, and/or language deficits. There are three proposed mechanisms by which an expanded *C9orf72* repeat might act: reduced gene expression, accumulation of RNA foci, and/or aggregation of dipeptide repeat proteins [1, 3–7].

Although methylation of the *C9orf72* promoter, expression levels of *C9orf72* transcripts, and the length of the hexanucleotide expansion have been studied in blood [7–18], they have not been evaluated together in a single comprehensive large-scale study. Hence, we set out to perform a thorough characterization of our clinical cohort, which enabled us to examine correlations between these variables and to determine whether they are associated with features of *C9orf72*-linked diseases. Moreover, because we collected specimens longitudinally and from multiple family members, we were able to investigate changes over time and within families. Our extensive assessment of this cohort may help to improve our understanding of these complicated neurodegenerative disorders.

## Methods

### Participants

All biological specimens were collected at our ALS Center at Mayo Clinic Florida between 2008 and 2018. Our primary cohort consisted of 75 unrelated patients harboring a *C9orf72* repeat expansion (> 93% ALS), 33 ALS patients who did not carry the expansion, and 20 control subjects who had neither been diagnosed with ALS nor harbored a repeat expansion (*n* = 128; Table 1). We added 33 expansion carriers and 34 subjects without this expansion from *C9orf72* families to create our overall cohort (*n* = 195), containing 108 expansion carriers (Additional file 1: Table S1). Our overall cohort included 17 families with at least two members (*n* = 87). In addition, multiple time-points were collected for 35 *C9orf72* expansion carriers with up to seven time-points.

**Table 1:** Characteristics of primary cohort

Variable	C9Plus (*n* = 75)	C9Minus (*n* = 33)	Control (*n* = 20)
Sex, No. (% female)	41 (54.67)	15 (45.45)	10 (50.00)
Site of Onset, No. (% bulbar)	15 (20.00)	6 (18.18)	NA
Age at Collection, median (IQR), y	60.87 (55.28–65.94)	61.25 (56.58–65.05)	60.05 (52.05–65.26)
Age at Onset, median (IQR), y	59.25 (52.94–64.08)	60.08 (55.08–63.42)	NA
Survival after Onset^a^, median (IQR), y	1.98 (1.54–3.10)	1.99 (1.12–3.19)	NA
Methylation, median (IQR), %	4.05 (1.10–17.95)	0.24 (0.15–0.33)	0.46 (0.14–0.75)
Expression, median (IQR), %	73.32 (65.96–86.20)	110.70 (93.68–132.88)	100.00 (87.48–111.27)
Repeat Length, median (IQR), kb	20.05 (15.58–26.27)	NA	NA

*C9Plus* patients with a *C9orf72* repeat expansion, *C9Minus* patients without this expansion, *Control* control subjects without a neurodegenerative disease, *IQR* interquartile range, *NA* not applicable

^a^In total, 23 of our 75 affected *C9orf72* expansion carriers are currently alive (31%). Of the 33 patients without this expansion, 20 are alive (61%)

### *C9orf72* methylation

For our overall cohort (n = 195), DNA was extracted from blood using the Gentra Puregene Kit (Qiagen). Additionally, for a subset of individuals (*n* = 14), DNA was extracted from the frontal cortex and cerebellum with standard phenol/chloroform procedures. To determine methylation levels of the *C9orf72* promoter, a quantitative assay was performed as described previously [11]. In brief, 100 ng of genomic DNA was digested for 16 h with 2 units of both *HhaI* and *HaeIII* (New England BioLabs; experimental condition) or with 2 units of *HaeIII* (control condition), followed by heat inactivation. A quantitative real-time PCR was done on a Quantstudio 7 (Applied Biosystems) and methylation levels were estimated.

### *C9orf72* expression

Digital molecular barcoding was carried out on the nCounter system (NanoString Technologies) [19]. Briefly, for our expression cohort (*n* = 87), RNA was extracted from blood using the PAXgene Blood RNA Kit (PreAnalytiX) and the quality was determined on a 2100 Bioanalyzer (Agilent Technologies). Subsequently, 250 ng was used to assess total *C9orf72* transcripts, variant 1 transcripts (NM_145005.6), variant 2 transcripts (NM_018325.4), and intron containing transcripts (intron 1a [upstream of the repeat] and intron 1b [downstream of the repeat]). Hypoxanthine phosphoribosyltransferase 1 (*HPRT1*) and tyrosine 3-monooxygenase/tryptophan 5-monooxygenase activation protein zeta (*YWHAZ*) were used as endogenous controls.

Additionally, for a specific family (PED1) and matched controls, gene expression assays (TaqMan) were performed [19]. Approximately 200 ng of template RNA was used to generate complimentary DNA (cDNA) with the SuperScript III Kit (Invitrogen). Quantitative real-time PCR was then undertaken on a Quantstudio 7 for total *C9orf72* transcripts (Hs00376619_m1), *C9orf72* variant 1 (custom assay), and *C9orf72* variant 2 (custom assay), using *HPRT1* (Hs02800695_m1) and *YWHAZ* (Hs00852925_sH) as endogenous controls.

### *C9orf72* repeat length

High-quality DNA from blood was available for *C9orf72* expansion carriers (*n* = 97) from our overall cohort; DNA from the frontal cortex and cerebellum was obtained for a subset of individuals (*n* = 14). Southern blotting was performed as described elsewhere [14]. In short, approximately 10 μg of genomic DNA was digested with restriction enzyme *XbaI* (Promega Corporation), electrophoresed, transferred to a positively charged nylon membrane (Roche), and crosslinked via ultraviolet irradiation. After pre-hybridization, the membrane was hybridized with a digoxigenin (DIG)-labelled probe (Roche). To reduce the background signal, stringency washes and subsequent blocking were performed. An anti-DIG antibody (1:10,000; Roche) was utilized to allow visualization on autoradiography film. Expansion sizes were estimated using AlphaEase FC (Alpha Innotech).

### Statistical analysis

The three primary measures of this study (methylation levels, expression levels, and repeat lengths) were compared between groups using a Kruskal-Wallis rank sum test, a Wilcoxon rank sum test, or a paired Wilcoxon signed-rank test, as appropriate for a given comparison. Correlations between continuous variables were assessed using a Spearman’s test of correlation; Spearman’s correlation coefficient *r* was estimated. A linear regression model was used with repeat length as outcome to determine the presence of an association with the disease status (affected versus unaffected), when adjusting for age at collection. Cox proportional hazards regression models were used to examine associations between the three primary measures and survival after onset, where measures were dichotomized using the median, models were adjusted for age at onset, and censoring occurred at the date of last follow-up. Changes in the three primary measures over time were examined utilizing mixed effects linear regression models, including a fixed effect for time and a random effect for each individual. When more than one measurement was available for a subject and/or time-point, the mean of those measurements was used. To adjust for multiple testing, a Bonferroni correction was utilized, separately for each group of similar statistical tests. All statistical tests were two-sided and were performed using R (v3.5.3).

## Results

### Hypermethylation of *C9orf72* promoter

**Fig. 1:**
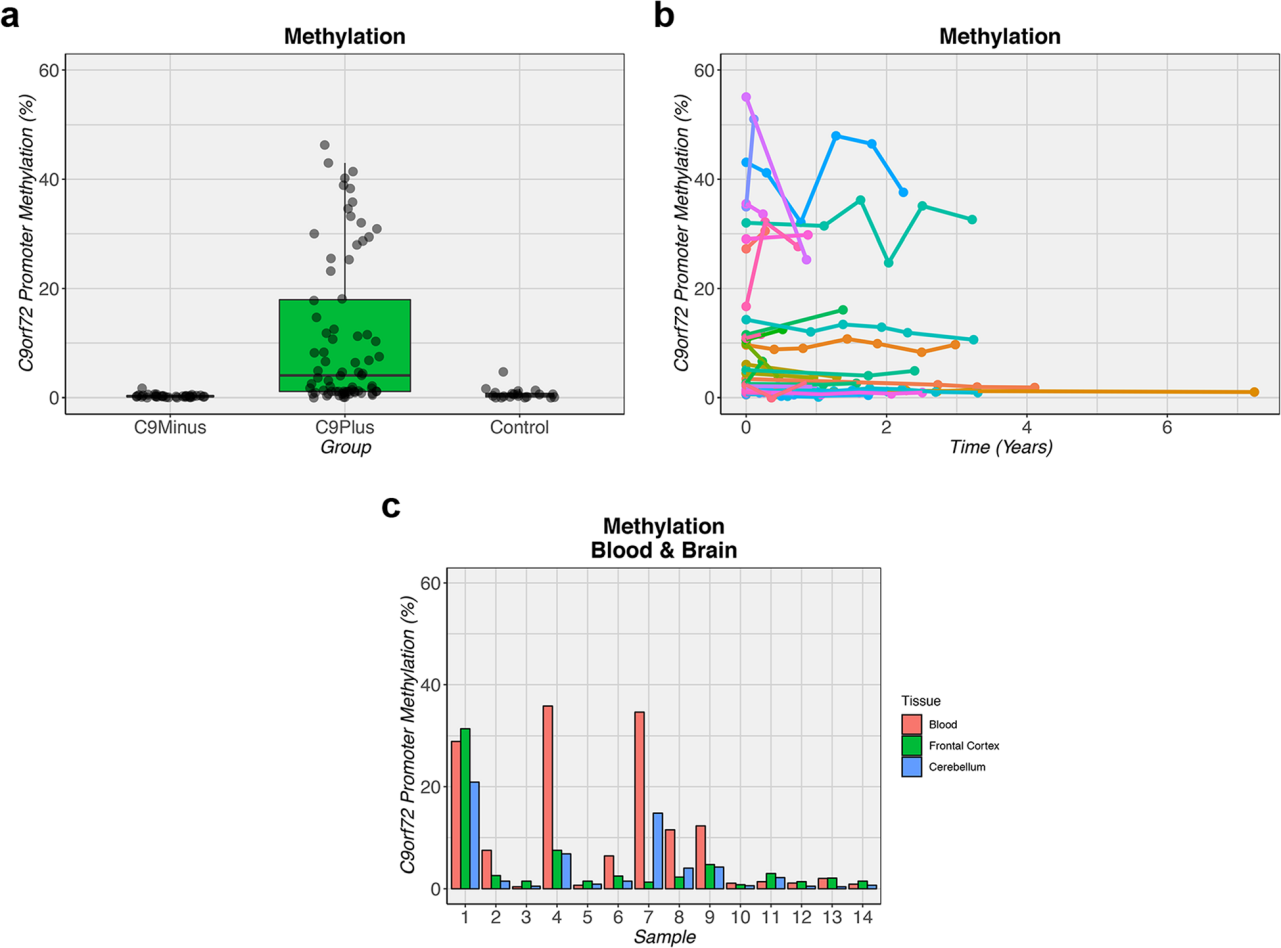
Methylation of *C9orf72* promoter. **a** Increased methylation levels of the *C9orf72* promoter are observed when comparing patients with a *C9orf72* repeat expansion (C9Plus) to patients without this expansion (C9Minus) or to control subjects (Control). The median is represented by a solid black line, and each box spans the interquartile range (IQR; 25th percentile to 75th percentile). **b** Methylation levels appear to be fairly stable over time: subjects with high methylation levels remain high, while those with low levels remain low. For each individual, longitudinal measurements are connected by a solid colored line. **c** In blood, frontal cortex, and cerebellum, a similar methylation pattern is seen. For each individual, three bars are displayed that correspond to methylation levels in a specific tissue type

Methylation levels of the *C9orf72* promoter were determined in blood for our primary cohort (Table 1). A significant difference in methylation levels was observed between groups (*p* = 4.5e-15; Table 2). *C9orf72* expansion carriers had median methylation levels of 4.1%, which was significantly higher than non-expansion carriers (0.2%, *p* = 1.7e-13) and controls (0.5%, *p* = 3.3e-07; Fig. 1a). With increasing methylation levels, there was a decrease in expression levels of total *C9orf72* transcripts (*r*: - 0.42, *p* = 5.5e-05), variant 1 transcripts (*r*: - 0.35, *p* = 9.5e-04), variant 2 transcripts (*r*: - 0.35, *p* = 8.7e-04), and intron 1b containing transcripts (*r*: - 0.30, *p* = 0.005). Within the subset of individuals with a *C9orf72* repeat expansion, we found hypermethylation in 36.0%, when using a threshold of 10.0% [11]. Similar findings were obtained in our overall cohort that included additional members from *C9orf72* families (Additional file 1**:** Table S1). We did not detect a significant difference between *C9orf72* expansion carriers with or without symptoms (*p* = 0.17).

**Table 2:** Methylation and expression levels of *C9orf72*

	Groups			*P*-Values			
Variable	C9Plus	C9Minus	Control	Groups^a^	C9Plus vs C9Minus^b^	C9Plus vs Control^b^	C9Minus vs Control^b^
Methylation, median (IQR), %	4.05 (1.10–17.95)	0.24 (0.15–0.33)	0.46 (0.14–0.75)	4.45e-15	1.70e-13	3.33e-07	0.17
Total, median (IQR), %	73.32 (65.96–86.20)	110.70 (93.68–132.88)	100.00 (87.48–111.27)	2.06e-07	3.67e-07	9.08e-05	0.13
Variant 1, median (IQR), %	55.66 (48.14–72.82)	80.10 (70.31–94.64)	100.00 (74.87–110.67)	1.07e-05	2.83e-04	3.65e-05	0.06
Variant 2, median (IQR), %	61.97 (55.23–73.67)	104.75 (85.86–139.38)	100.00 (90.04–120.12)	9.66e-08	3.43e-07	1.10e-05	0.76
Intron 1a, median (IQR), %	411.20 (244.09–637.62)	263.06 (103.15–398.10)	100.00 (37.09–290.55)	8.40e-04	0.01	4.03e-04	0.13
Intron 1b, median (IQR), %	61.18 (54.37–90.30)	98.61 (75.58–109.73)	100.00 (67.11–130.03)	0.003	0.002	0.01	0.48

*C9Plus* patients with a *C9orf72* repeat expansion, *C9Minus* patients without this expansion, *Control* control subjects without a neurodegenerative disease, *IQR* interquartile range

^a^A Kruskal-Wallis rank sum test is performed to determine whether a significant difference exists between groups for each of the six variables: levels of *C9orf72* promoter methylation (Methylation), total *C9orf72* transcripts (Total), variant 1 transcripts (Variant 1), variant 2 transcripts (Variant 2), intron 1a containing transcripts (Intron 1a), and intron 1b containing transcripts (Intron 1b; *p* < 0.008 is considered significant after Bonferroni correction)

^b^A Wilcoxon rank sum test is used when the Kruskal-Wallis test is significant for each of the three pairwise comparisons: C9Plus vs C9Minus, C9Plus vs Control, and C9Minus vs Control (*p* < 0.017 is considered significant after Bonferroni correction)

To determine whether methylation levels of the *C9orf72* promoter were stable over time, we subsequently assayed longitudinally collected blood specimens. Although some variability in promoter methylation over time was observed, subjects with low methylation levels remained low, while subjects with relatively high methylation levels remained high (*p* = 0.56; Fig. 1b). These findings were further substantiated by our assessment of *C9orf72* expansion carriers who had died and for whom brain tissue was available (*n* = 14): methylation levels in blood correlated with those in the frontal cortex (*r*: 0.56, *p* = 0.04) or cerebellum (*r*: 0.81, *p* = 3.8e-04; Fig. 1c, Additional file 1: Figure S1a-b).

### Reduced expression of *C9orf72* transcripts

**Fig. 2:**
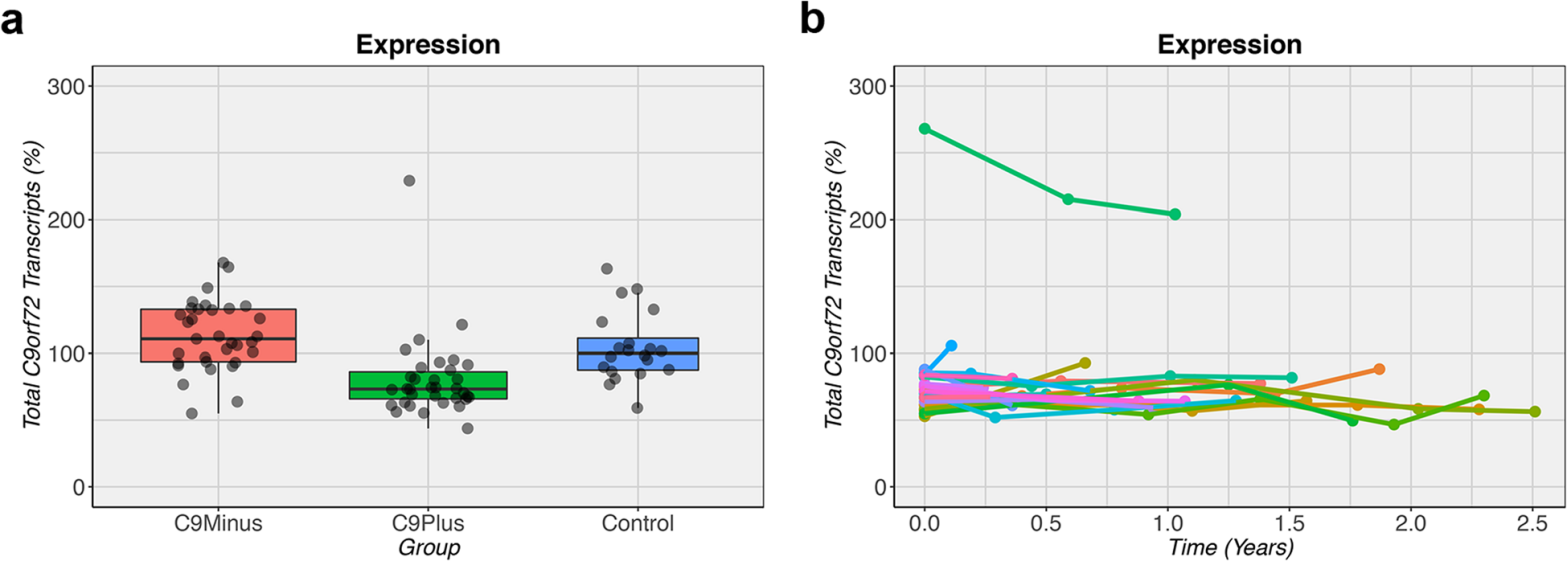
Expression of *C9orf72* transcripts. **a** In patients with an expanded *C9orf72* repeat (C9Plus), the expression levels of total *C9orf72* transcripts are lower than in patients without this expansion (C9Minus) or in control subjects (Control). The median is represented by a solid black line, and each box spans the interquartile range (IQR; 25th percentile to 75th percentile). **b** Over time, the levels of total *C9orf72* transcripts remain relatively stable. One outlier is detected with levels that are consistently higher than in other *C9orf72* expansion carriers. This outlier has an expansion of 7.9 kb (~ 900 repeats) in addition to an expansion of 3.4 kb (~ 200 repeats). For each individual, longitudinal measurements are connected by a solid colored line

RNA expression levels of *C9orf72* transcripts in blood were evaluated in our expression cohort (Additional file 1: Table S2). For total *C9orf72* transcripts, a significant difference was detected between groups (*p* = 2.1e-07; Table 2, Fig. 2a). Specifically, median expression levels were lower in *C9orf72* expansion carriers (73.3%) compared to non-expansion carriers (110.7%, *p* = 3.7e-07) and controls (100.0%, *p* = 9.1e-05). A similar pattern was seen for variant 1 transcripts, variant 2 transcripts, and intron 1b containing transcripts (*p* ≤ 0.003). Interestingly, intron 1a containing transcripts (*p* = 8.4e-04) demonstrated elevated levels in *C9orf72* expansion carriers when comparing them to (disease) controls (*p* ≤ 0.01; Table 2). For total *C9orf72* transcripts, no significant difference was observed between affected and unaffected expansion carriers (*p* = 0.63).

Assessment of longitudinal changes in RNA expression levels uncovered that most *C9orf72* expansion carriers had low expression levels in blood that were stable over time (*p* = 0.47; Fig. 2b). One individual, however, was consistently identified as an outlier with relatively high total *C9orf72* expression levels (> 200%). This individual was an otherwise typical ALS patient with an age at onset of 56 years and a survival of about 2 years after the onset of symptoms. The Southern blot of this person displayed two bands, including a weak band of 7.9 kb (~ 900 repeats) and a smaller, sharper band of 3.4 kb (~ 200 repeats; Additional file 1**:** Figure S2a).

### Instability of *C9orf72* repeat

**Fig. 3:**
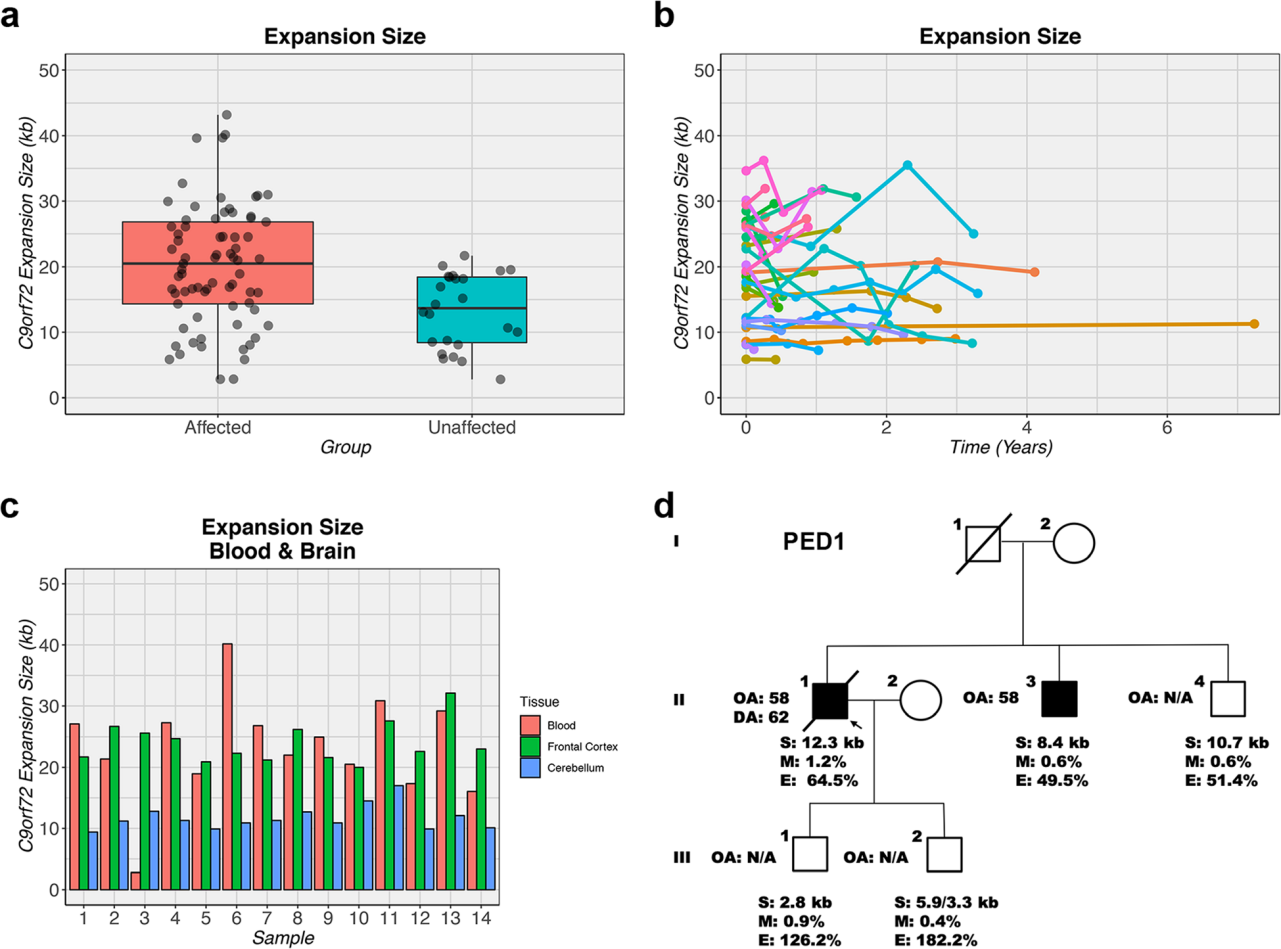
Expansion size of *C9orf72* repeat. **a** When comparing *C9orf72* expansion carriers with symptoms (affected) to those presently without symptoms (unaffected), a longer expansion is observed in affected subjects. The median is represented by a solid black line, and each box spans the interquartile range (IQR; 25th percentile to 75th percentile). **b** Fluctuations are detected in the expansion size of subjects with an expanded *C9orf72* repeat; no clear pattern emerges. For each individual, longitudinal measurements are connected by a solid colored line. **c** There is no correlation between expansion sizes in blood, frontal cortex, or cerebellum. For each individual, three bars are displayed that correspond to expansion sizes in a specific tissue type. **d** One pedigree is shown (PED1) with the proband (II-1; arrow), his siblings (II-3 and II-4), and his children (III-1 and III-2). Affected individuals are denoted by a solid black square. Information is provided regarding the onset age (OA), death age (DA), expansion size (S), methylation level (M), and expression level (E), when available (N/A)

We estimated the repeat length of 64 *C9orf72* expansion carriers in our primary cohort for whom sufficient high-quality DNA was available from blood. In these expansion carriers, the median repeat length was 20.1 kb (~ 3000 repeats). Additionally, we included 33 extra expansion carriers from our overall cohort, resulting in a median repeat length of 18.2 kb (**~** 2700 repeats). Importantly, when comparing *C9orf72* expansion carriers who were affected (*n* = 73) to expansion carriers who were not (yet) affected (*n* = 24), a significant difference was observed: affected expansion carriers had a repeat length of 20.5 kb (**~** 3000 repeats) versus 13.7 kb (**~** 1900 repeats) in unaffected expansion carriers (*p* = 8.9e-04; Fig. 3a). To determine whether this association could have been driven by a difference in age at collection, we performed a linear regression analysis with repeat length as outcome. When including the disease status (affected versus unaffected) as a single variable, a significant association was detected (*p* = 0.001). This association remained significant (*p* = 0.01), after adding age at collection to our model (*p* = 0.80). We did not detect a significant correlation between the repeat length and methylation or expression levels (*p* ≥ 0.31).

Next, we examined blood specimens collected at multiple time-points. In individual cases, the length did vary; however, in general, the repeat length did not change over time (*p* = 0.50; Fig. 3b). One individual, for instance, exhibited a minor increase of 0.5 kb (~ 100 repeats) over a period of more than 7 years (Additional file 1: Figure S2b). For a subset of *C9orf72* expansion carriers (*n* = 14), repeat lengths were also estimated in autopsy tissues, which did not uncover a correlation between lengths in blood and brain (*p* ≥ 0.44; Fig. 3c, Additional file 1: Figure S1c-d).

### Detection of clinical associations

We restricted our analysis to unrelated patients harboring an expanded *C9orf72* repeat to evaluate associations with clinical features, including age at onset, age at collection, site of onset, sex, and survival after onset. After adjustment for multiple testing, no significant associations were detected with methylation levels, expression levels, or repeat lengths (data not shown). When we added affected (*n* = 9) and unaffected (*n* = 24) family members carrying the expansion, however, we did detect a significant association between the length of the repeat and age at collection (*r*: 0.27, *p* = 0.008), indicating that smaller expansions are present in younger individuals.

### Description of families

**Fig. 4:**
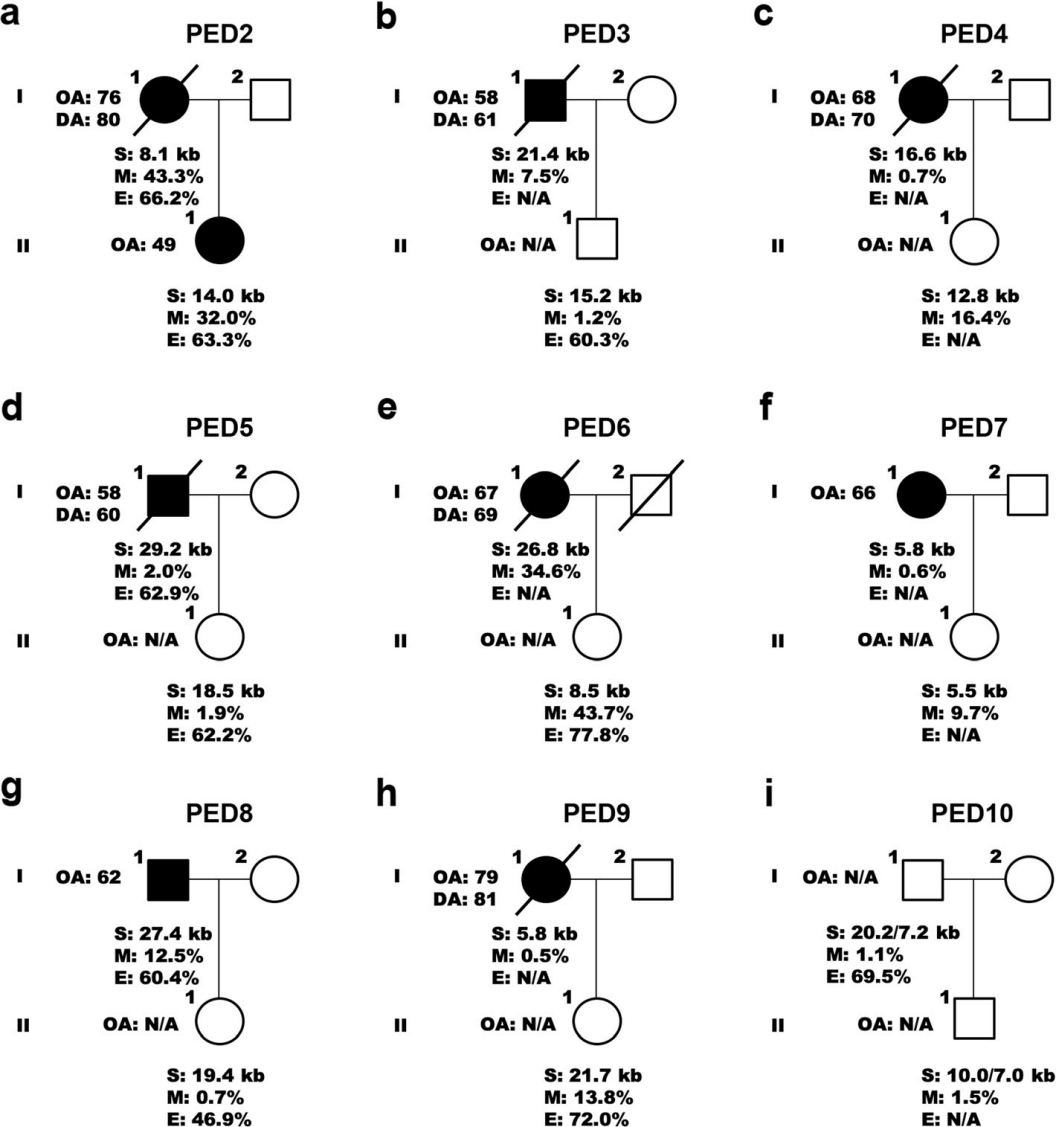
Parent-offspring transmissions I. **a – i** This figure contains nine parent-offspring transmissions (PED2 to PED10). The last two transmissions (PED9 and PED10) occurred in a different branch of the same family. Affected individuals are denoted by a solid black circle or square. Information is provided regarding the onset age (OA), death age (DA), expansion size (S), methylation level (M), and expression level (E), when available (N/A)

**Fig. 5:**
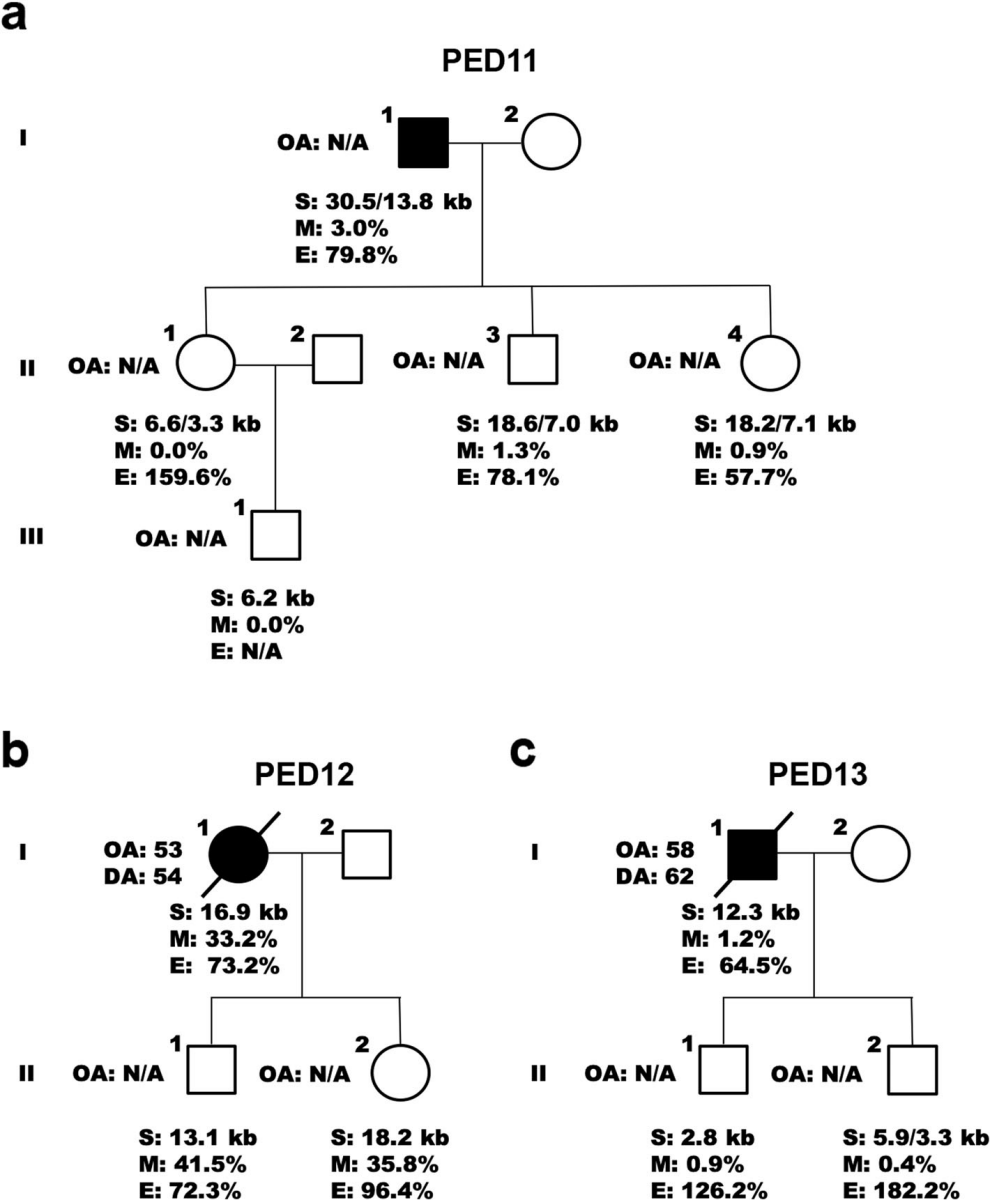
Parent-offspring transmissions II. **a – c** Three pedigrees (PED11 to PED13) are displayed with additional parent-offspring transmissions (eight in total). The first pedigree (PED11) represents another branch from a family with multiple branches (PED9 and PED10; Fig. 4h-i). The last pedigree (PED13) is a simplified version of a pedigree shown elsewhere (PED1; Fig. 3d). Affected individuals are denoted by a solid black circle or square. Information is provided regarding the onset age (OA), death age (DA), expansion size (S), methylation level (M), and expression level (E), when available (N/A)

To examine changes within families, we then compared methylation levels, expression levels, and repeat lengths in parent-offspring transmissions. In 14 out of 17 transmissions (82.4%) from 10 unrelated families, hypermethylation was either present or absent (Fig. 4, Fig. 5). Of the remaining transmissions, two (11.8%) demonstrated a change in status from lowly methylated to hypermethylated and one (5.9%) from hypermethylated to lowly methylated. In seven out of 10 transmissions where RNA was available (70.0%), expression levels appeared to be relatively stable. In the remaining three transmissions (30.0%), we detected an elevation in transcript levels of children with an expansion size of less than 5 kb (Fig. 5a, Fig. 5c). Interestingly, contracted expansions were commonly encountered and present in 10 out of 17 parent-to-child transmissions (58.8%; Fig. 4, Fig. 5, and Additional file 1: Figure S2c); all these transmissions exhibited a difference of more than 5 kb between generations and the majority were paternal (90.0%). An increase in expansion size was less common and seen in two maternal transmissions (11.8%), while a stable expansion was observed in five maternal transmissions (29.4%). When comparing expansion sizes between parents and children from these 17 parent-offspring pairs, the expansion size was significantly longer in parents than in children (*p* = 0.01). Similar findings were obtained when restricting the analysis to one parent-to-child transmission per parent (*n* = 12).

One of the families with a contracted expansion, PED1, demonstrated changes between the proband (II-1), his siblings (II-3 and II-4), and his children (III-1 and III-2; Fig. 3d). The proband had been diagnosed with ALS in his late fifties and died after a disease duration of approximately 4 years. One of his siblings also developed ALS in his fifties, while another sibling was currently unaffected, just like his children who were relatively young (in their thirties). When examining methylation levels of the *C9orf72* promoter in this family, low levels were observed in all family members. Expression levels, however, differed: the proband had a total *C9orf72* transcript level of 64.5%, his siblings of 49.5 and 51.4%, and his children of 126.2 and 182.2%, when comparing them to controls matched based on sex and age (100.0%). Examination of the repeat length revealed that the proband harbored an expansion of 12.3 kb (~ 1700 repeats), his affected sibling of 8.4 kb (~ 1000 repeats), and his unaffected sibling of 10.7 kb (~ 1400 repeats). His children were found to carry a contracted expansion with repeat lengths of 2.8 kb (~ 100 repeats) and 5.9/3.3 kb (~ 600/200 repeats), respectively (Additional file 1: Figure S2d).

## Discussion

Our characterization of *C9orf72* expansion carriers in blood showed that 36% demonstrate hypermethylation of the *C9orf72* promoter. Additionally, we detected reduced expression levels of *C9orf72* transcripts and, when comparing expansion sizes between affected and unaffected individuals, we found a difference of roughly 7 kb (> 1000 repeats). Investigation of longitudinal changes uncovered relatively stable methylation and expression levels; however, fluctuations were seen in the size of the expansion. Similarly, in parent-offspring transmissions, little variation was observed in methylation and expression, whereas instability was detected in the length of the repeat, which contracted in the majority of cases.

The hypermethylation frequency we detected is in agreement with previous studies that investigated blood from ALS patients [8, 11]. We also discovered that the methylation status was fairly stable over time and within families. These findings were substantiated by our detection of correlations between methylation levels in blood and brain, confirming other reports [8, 11]. The *C9orf72* expression pattern we found in blood was similar to that described in the frontal cortex as well [19]: in both regions, we demonstrated a decrease in most *C9orf72* transcripts. Additionally, we noticed an upregulation of transcripts containing intron 1a, an intronic area located just before the repeat. While transcripts containing intron 1b (after the repeat) were reduced, this seems to indicate that the expanded repeat might trigger the production of abortive transcripts [19, 20] and/or a switch in transcription start site usage [21]. Intriguingly, in individuals with small repeat sizes (< 5 kb), we detected relatively high *C9orf72* expression levels, including an outlier with expression levels above 200%. These findings are in accordance with other descriptions of subjects with intermediate or small expansions who exhibit normal to elevated expression levels [8, 22–24]. In general, though, we did not detect an association between the length of the *C9orf72* repeat expansion and methylation or expression levels, which contradicts other reports [11].

Importantly, our examination of expansion sizes in blood revealed a pattern different from that seen in brain tissue [14]. Individual cases where discrepancies were noted between sizes in blood and brain have already been described by us and others [14, 16, 22, 25]. Together, these findings suggest that repeat lengths measured in blood, an unaffected region, might not mirror lengths in the central nervous system, possibly due to somatic mosaicism. Despite the fact that blood can be useful in establishing the presence or absence of *C9orf72* repeat expansions, our findings indicate that one should be careful when interpreting size estimates based on blood measurements.

In several repeat expansion disorders, such as myotonic dystrophy [26], anticipation has been described where a more severe phenotype is thought to arise from an increase in the length of the repeat in successive generations. One study suggested that the age at onset in patients with an expanded *C9orf72* repeat decreases from one generation to the next [27]. These findings align with another report that detected an increase in expansion size when transmitting the repeat from parent to child [18]. Although these studies could indeed point to anticipation, our work suggests that this may not be the case. In successive generations, we actually noticed that the repeat expansion had a tendency to contract, which was also reported by others [12]. One wonders, therefore, whether the earlier age at onset reported in the former study might be a reflection of selection bias, recall bias, and/or diagnostic bias. Furthermore, it seems plausible that, when a disease runs in a given family, this could raise awareness in family members who may seek medical attention sooner and who may receive a diagnosis at an earlier age. Additionally, the increase in expansion size observed in the latter study depends on one parent-to-child transmission. The relatively high frequency of contractions we detected in a larger number of parent-offspring transmissions, mainly in paternal transmissions, is similar to that described in Friedreich’s ataxia where paternally inherited expansions generally decrease in size [28]. Based on our observation that testes can contain a small contracted expansion in addition to a long expansion [14], we hypothesize that the presence of such a contraction in male germ cells might explain why contractions are primarily seen in paternal transmissions. Notably, it is possible that expansions are relatively stable in germ cells, whereas they demonstrate instability in somatic cells. The biological relevance of contractions remains unclear and should be investigated in future studies.

Our discovery of contracted expansions is supported by our detection of significantly smaller expansions in presently unaffected individuals than in affected individuals. Moreover, it also agrees with the association we found between the length of the expansion and age at collection in our extended cohort of *C9orf72* expansion carriers. Basically, these findings suggest that contractions occur frequently and that, as a consequence, the repeat length is smaller in young expansion carriers who are currently unaffected. It should be noted, though, that the association with disease status (affected versus unaffected) seemed more robust than that with age at collection. Over time, the length of the expansion may fluctuate, exhibiting both contractions and expansions. This instability could result in profound differences in repeat length, and hence, the length in blood at a given point in time may be a poor reflection of the original length. As such, this could explain conflicting results reported in the literature, depending on the time-points or tissues analyzed as well as the number of unaffected individuals studied [11, 12, 14, 17].

### Limitations

Although we performed a thorough characterization of our clinical cohort of subjects harboring an expanded *C9orf72* repeat, we realize that the methods we employed and the specimens we collected have limitations. The methylation-sensitive restriction enzyme-based assay we utilized, for instance, depends on the methylation status of a single CpG. Despite the fact that this assay has been validated using bisulfite sequencing and has been shown to provide a good estimate of the entire promoter area [11], we cannot rule out the possibility that other CpG sites are methylated. Because RNA was not available for all subjects included in our study, we could only obtain *C9orf72* transcript levels from a subset of individuals. It should also be noted that *C9orf72* transcript variant 3 was not assessed, since it cannot be detected reliably using digital molecular barcoding [19]. Additionally, we acknowledge that Southern blots are challenging and that the presence of a smear, frequently detected in blood, can hamper its accuracy. To improve our estimates, we did measure our samples multiple times (2.4x, on average) and the degree of variability in repeat lengths was relatively low (median subject-specific standard deviation: 1.9 kb [~ 300 repeats]). Because we focused on blood, we cannot exclude the possibility that our findings would have been different if we had evaluated an affected region. Given the limited availability of brain tissue from multiple generations, for example, it is currently unclear whether the contractions we detected in blood can be observed in brain tissue. It should also be stressed that due to the relatively short survival after onset of ALS patients, it is difficult to collect longitudinal samples from affected individuals over an extended period of time, which might have influenced our ability to detect significant changes over time. Lastly, our study mainly included ALS patients, and therefore, additional studies will have to be performed to determine whether similar findings can be obtained in FTD patients.

## Conclusions

In this comprehensive blood-based study, we evaluated the methylation status, expression level, and repeat length in a clinical cohort of *C9orf72* expansion carriers. We detected hypermethylation of the *C9orf72* promoter and reduced expression levels of *C9orf72* transcripts, which were both stable over time and within families. The length of the repeat, on the other hand, demonstrated more variability and was not comparable to that detected in other regions; in parent-to-child transmissions, contractions were more commonly encountered than expansions, arguing against anticipation in *C9orf72*-linked diseases.

## Supplementary information

**Additional file 1 Table S1**. Characteristics of overall cohort; **Table S2**. Characteristics of expression cohort; **Figure S1**. Comparison blood and brain; **Figure S2**. Southern blot examples.

## Data Availability

All data relevant to the study are included in the article or as supplementary information. Upon reasonable request, additional information (e.g., protocols) will be shared by the corresponding authors.

## Abbreviations

ALS: Amyotrophic lateral sclerosis
C9orf72: C9orf72-SMCR8 complex subunit
DIG: Digoxigenin
FTD: Frontotemporal dementia
HPRT1: Hypoxanthine phosphoribosyltransferase 1
YWHAZ: Tyrosine 3-monooxygenase/tryptophan 5-monooxygenase activation protein zeta
