# Supplementary material for: Elevated methylation levels, reduced expression levels, and frequent contractions in a clinical cohort of C9orf72 expansion carriers
Source: Mol Neurodegener. 2020 Jan 30;15:7. doi: 10.1186/s13024-020-0359-8 (PMC6993399; doi:10.1186/s13024-020-0359-8)
Supplement: Supplementary file 1 — Additional file 1 Table S1. Characteristics of overall cohort; Table S2. Characteristics of expression cohort; Figure S1. Comparison blood and brain; Figure S2. Southern blot examples. [file 13024_2020_359_MOESM1_ESM.docx]

**Additional File 1**

**Elevated methylation levels, reduced expression levels, and frequent contractions in a clinical cohort of *C9orf72* expansion carriers**

**Jazmyne L. Jackson^1^, NiCole A. Finch^1^, Matthew C. Baker^1^, Jennifer M. Kachergus^2^, Mariely DeJesus-Hernandez^1^, Kimberly Pereira^1^, Elizabeth Christopher^1^, Mercedes Prudencio^1^, Michael G. Heckman^3^, E. Aubrey Thompson^2^, Dennis W. Dickson^1^, Jaimin Shah^4^, Björn Oskarsson^4^, Leonard Petrucelli^1^, Rosa Rademakers^1^, and Marka van Blitterswijk^1^**

**Affiliations:**

1. **Department of Neuroscience, Mayo Clinic, Jacksonville, Florida, USA**
2. **Department of Cancer Biology, Mayo Clinic, Jacksonville, Florida, USA**
3. **Division of Biomedical Statistics and Informatics, Mayo Clinic, Jacksonville, Florida, USA**
4. **Department of Neurology, Mayo Clinic, Jacksonville, Florida, USA**

**Contents:**

Table S1. Characteristics of overall cohort

Table S2. Characteristics of expression cohort

Figure S1. Comparison blood and brain

Figure S2. Southern blot examples

**Table S1. Characteristics of overall cohort**

| Variable | C9Plus (n=108)^a^ | C9Minus (n=37)^a^ | Control (n=50)^a^ |
| --- | --- | --- | --- |
| Sex, No. (% female) | 59 (54.63) | 17 (45.95) | 30 (60.00) |
| Site of Onset, No. (% bulbar) | 16 (14.81) | 6 (16.22) | NA |
| Age at Collection, median (IQR), y | 58.53 (48.39–64.83) | 61.25 (56.75–65.36) | 47.35 (36.09–62.64) |
| Age at Onset, median (IQR), y | 59.25 (53.25–63.95) | 59.58 (55.10–63.42) | NA |
| Survival after Onset^b^, median (IQR), y | 1.98 (1.44–3.13) | 1.96 (1.10–3.18) | NA |
| Methylation, median (IQR), % | 3.05 (1.10–15.12) | 0.23 (0.11–0.31) | 0.22 (0.09–0.60) |
| Expression, median (IQR), % | 74.11 (66.30–83.77) | 110.62 (94.41–135.67) | 100.00 (89.72–110.48) |
| Repeat Length, median (IQR), kb | 18.17 (11.16–24.51) | NA | NA |

C9Plus: individuals carrying an expanded *C9orf72* repeat; C9Minus: patients without this expansion; Control: control subjects without a neurodegenerative disease; IQR: interquartile range; NA: not applicable.

^a^Of the family members included in this cohort (n=67), 33 carry a *C9orf72* repeat expansion and are added to the C9Plus group (n=108). Four of the family members without a *C9orf72* repeat expansion are affected by a neurodegenerative disease (dementia) and/or represent a phenocopy; those individuals are added to the C9Minus group (n=37). The remaining 30 family members are unaffected and added to the Control group (n=50).

^b^In total, 27 of our 84 affected *C9orf72* expansion carriers are currently alive (32%). Of the 33 patients without this expansion, 20 are alive (61%).

**Table S2. Characteristics of expression cohort**

| Variable | C9Plus (n=34) | C9Minus (n=33) | Control (n=20) |
| --- | --- | --- | --- |
| Sex, No. (% female) | 16 (47.06) | 15 (45.45) | 10 (50.00) |
| Site of Onset, No. (% bulbar) | 6 (17.65) | 6 (18.18) | NA |
| Age at Collection, median (IQR), y | 60.52 (56.98–63.07) | 61.25 (56.58–65.05) | 60.05 (52.05–65.26) |
| Age at Onset, median (IQR), y | 59.25 (54.90–61.73) | 60.08 (55.08–63.42) | NA |
| Survival after Onset^a^, median (IQR), y | 2.16 (1.59–4.05) | 1.99 (1.12–3.19) | NA |
| Methylation, median (IQR), % | 3.04 (1.09–14.16) | 0.24 (0.15–0.33) | 0.46 (0.14–0.75) |
| Expression, median (IQR), % | 73.32 (65.96–86.20) | 110.70 (93.68–132.88) | 100.00 (87.48–111.27) |
| Repeat Length, median (IQR), kb | 19.26 (15.54–24.93) | NA | NA |

C9Plus: patients with a *C9orf72* repeat expansion; C9Minus: patients without this expansion; Control: control subjects without a neurodegenerative disease; IQR; interquartile range; NA: not applicable.

^a^In total, 8 of our 34 affected *C9orf72* expansion carriers are currently alive (24%). Of the 33 patients without this expansion, 20 are alive (61%).

**
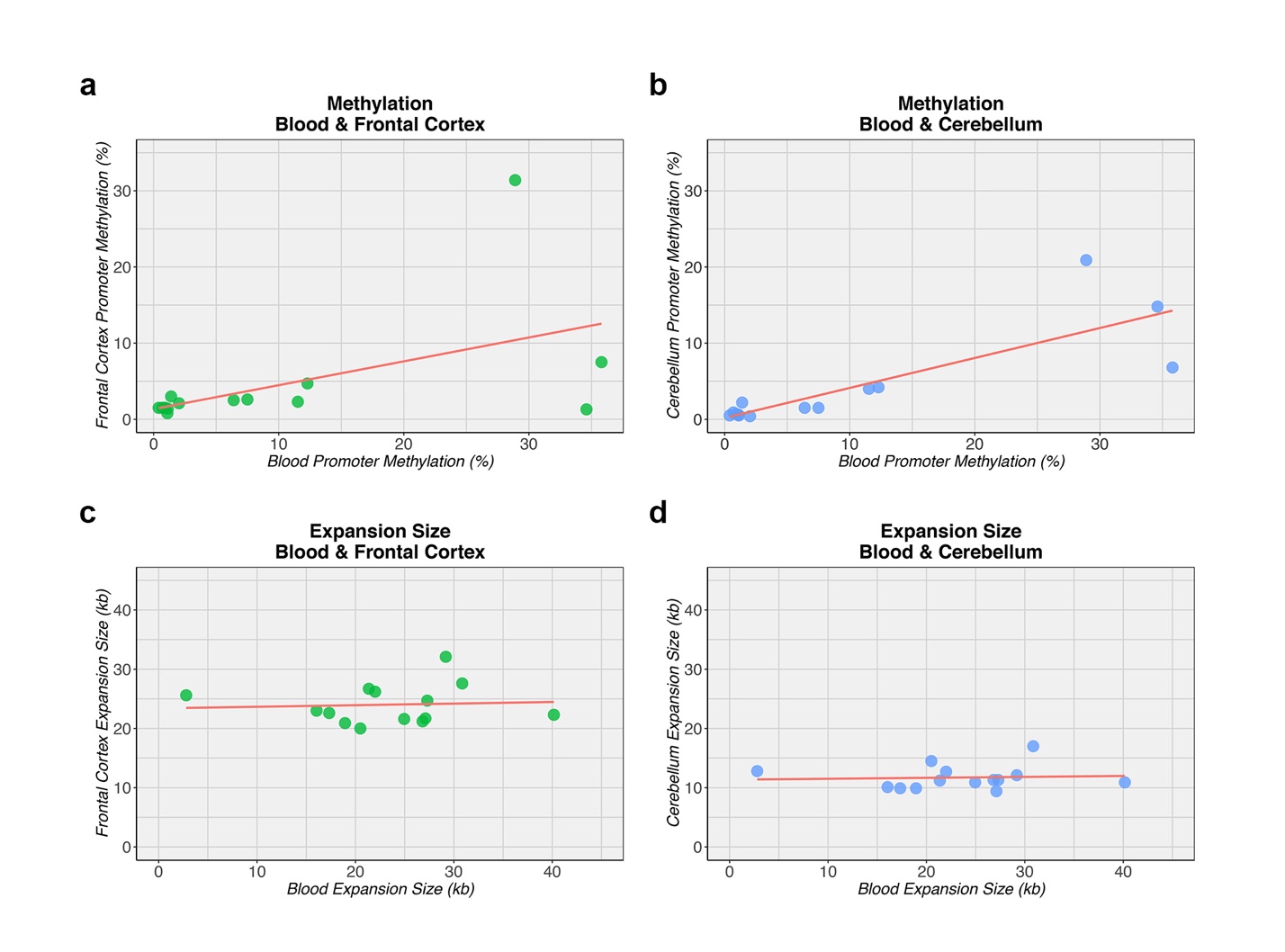
Figure S1. Comparison blood and brain**

**a-b** Higher *C9orf72* promoter methylation levels in blood are correlated with higher methylation levels in the frontal cortex (left) and cerebellum (right). **c-d** There is no correlation between the *C9orf72* expansion size in blood and the frontal cortex (left) or cerebellum (right). In these plots, the solid red line is the linear regression line. Each solid circle represents a sample from the frontal cortex (green) or cerebellum (blue).

**Figure S2. Southern blot examples**

**
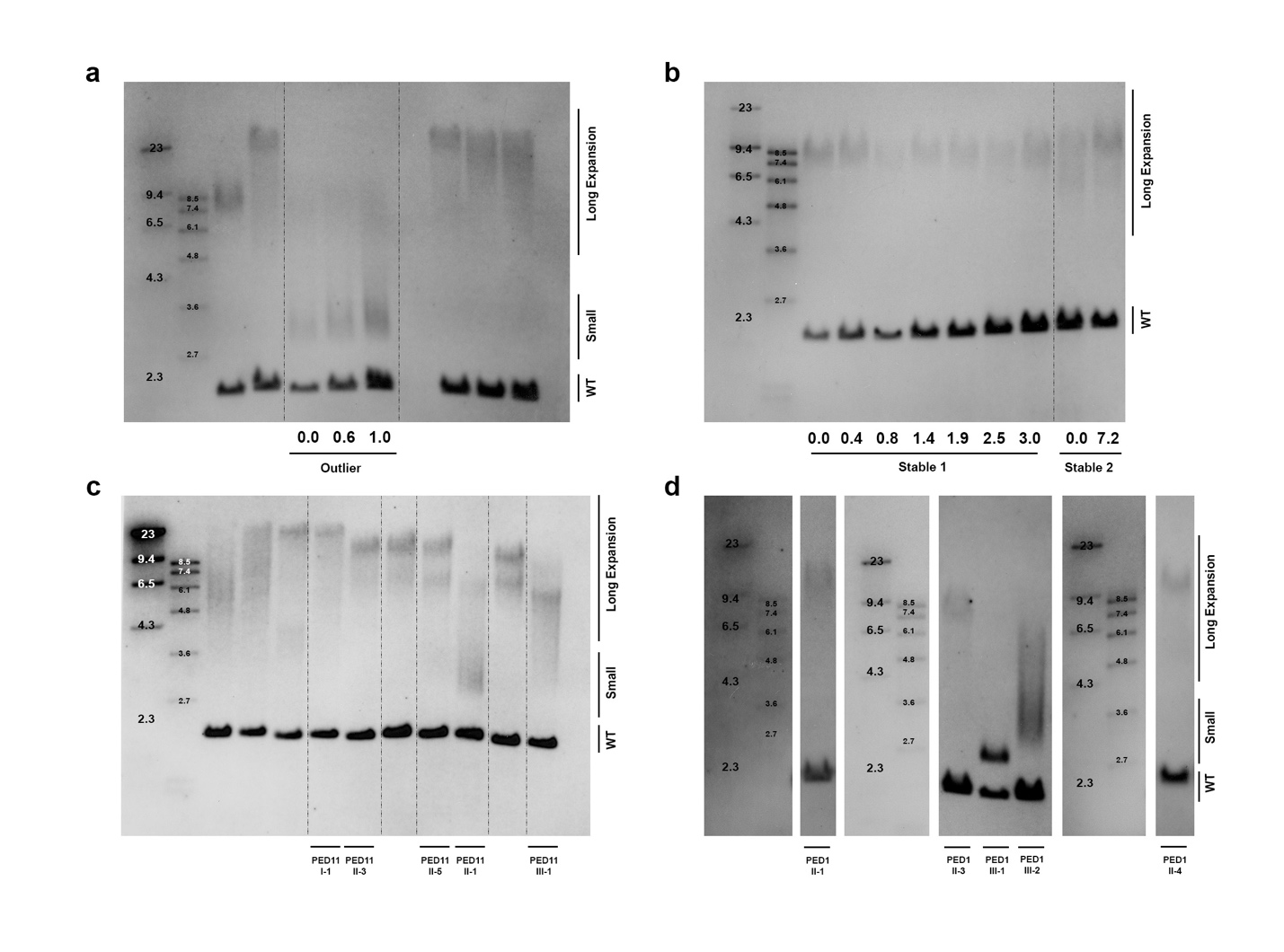
**

**a** The first panel displays the outlier described in our main text with high *C9orf72* expression levels (>200%). In addition to the wild-type allele (WT), two bands are shown for time-points that span a period of 1 year: one weak band of 7.9 kb and a strong band of 3.4 kb. **b** In the second panel, two representative examples are shared of subjects with relatively long expansions that are stable over time (up to 7.2 years). **c** The third panel contains several members of a large family; for simplicity, only individuals from one branch (PED11; **Figure 5a**) are specified. Multiple bands are present and parent-offspring transmissions result in contractions. **d** In the last panel, members of another family (PED1; **Figure 3d**) are displayed, including the proband, his siblings, and his children. In this family, contractions are also encountered in parent-to-child transmissions. Of note, in these panels, dashed lines are added as visual aids. In general, Southern blot estimates are based on the most abundant expansion size (peak), as determined by densitometry. When multiple bands are detected in a single individual, the repeat length of the highest band (longest expansion) is used in statistical analyses.
